# Wechselwirkungen von radikaler Prostatovesikulektomie und Diagnostik des Prostatakarzinoms: Eine medizinhistorische Bestandsaufnahme anlässlich 20 Jahre robotisch assistierter Therapie

**DOI:** 10.1007/s00120-020-01389-1

**Published:** 2020-11-17

**Authors:** Wolfgang Otto, Wolf F. Wieland

**Affiliations:** 1 Lehrstuhl für Urologie, Klinikum der Universität Regensburg, Franz-Josef-Strauß-Allee 11, 93053 Regensburg, Deutschland; 2 Urologie im Gesundheitsforum, Paracelsusstraße 2, 93053 Regensburg, Deutschland; 3 Ambulanz für Urologie, Nierenzentrum Eichstätt, Bahnhofsplatz 26, 85072 Eichstätt, Deutschland; 4 Bashkir State Medical University (BSMU), Ufa, Republic of Bashkortostan Russland; 5 People’s Friendship University of Russia (RUDN), Moscow, Russland

**Keywords:** Robotisch assistierte radikale Prostatovesikulektomie, Prostatabiopsie, Uropathologie, PSA, Skelettszintigraphie, Robotic-assisted radical prostatectomy, Prostate biopsy, Uropathology, Prostate specific antigen, Skeletal scintigraphy

## Abstract

Die Frage, was zuerst war – in diesem Falle die Diagnostik des Prostatakarzinoms oder seine Therapie – erscheint auf den ersten Blick widersinnig und erinnert an die klassische metapherartige Problemstellung, die schon den griechischen Schriftsteller Plutarch (45–125) beschäftigte. Ist es heute selbstverständlich, dass vor der Behandlung einer Erkrankung die sichere Diagnosestellung steht, so muss dies medizinhistorisch jedoch als nicht konsistent erachtet werden. Die Anfänge der radikalen Prostatektomie zur Behandlung des Prostatakarzinoms lassen sich, ähnlich wie die ersten operativen Therapien von Nieren- und Harnblasentumoren, in der Pionierzeit der Organchirurgie im Deutschen Kaiserreich (1871–1918) verorten. Die Etablierung dieses Eingriffs in seiner heutigen Form mit größeren Fallzahlen ist wiederum dem Nestor der US-amerikanischen Urologie, Hugh Hampton Young (1870–1945), zu verdanken, der 1904 die erste aus heutiger Sicht als vollumfänglich zu bezeichnende perineale Prostatovesikulektomie durchführte. Wenngleich die Indikation seither weitgehend unverändert geblieben ist, war dieser Eingriff in den letzten Jahrzehnten doch umfangreichen Veränderungen unterworfen. Wie aber hat sich die Diagnostik des Prostatakarzinoms in dieser Zeitspanne entwickelt? Naturgemäß sehr viel dynamischer! Denn als der Leiteingriff Prostatovesikulektomie bereits etabliert war, begann im Laufe des 20. Jahrhunderts erst langsam, dann dynamischer deren Entwicklung. Wir stellen anhand medizin(histor)ischer Originalquellen daher nicht nur die Grundlagen und Weiterentwicklungen des etablierten und zugleich immer wieder Innovationen unterworfenen Leiteingriffs der Urologie vor, sondern gehen vielmehr auch auf wesentliche Umfeldentwicklungen benachbarter medizinischer Disziplinen ein. Erst diese Entwicklungen schafften übrigens auch die Grundlage für die korrekte Indikationsstellung und das Aufzeigen von Alternativen zur radikalen Prostatovesikulektomie.

## Abstract (en)

The question of what came first—in this case the diagnosis of prostate cancer or its therapy—seems absurd at first glance and is reminiscent of the classic metaphor-like problem that preoccupied the Greek writer Plutarch (45–125). Today it is a matter of course that a reliable diagnosis is made before treating a disease, but this must be viewed as inconsistent in medical history. The beginnings of radical prostatectomy for the treatment of prostate cancer, like the first surgical therapies for kidney and bladder tumors, can be located in the pioneering period of organ surgery in the German Empire (1871–1918). The establishment of this procedure in its current form with larger numbers of cases is in turn thanks to the Nestor of American urology, Hugh Hampton Young, who carried out the first perineal prostatovesiculectomy, which from today’s perspective can be described as complete. Although the indication has remained largely unchanged since then, this intervention has undergone extensive changes in recent decades. But how has the diagnosis of prostate cancer developed in this period? Of course, much more dynamic. While the procedure prostatovesiculectomy was already established, development of prostate cancer diagnosis began first slowly in the course of the 20th century, then more dynamically. The following article uses medical (historical) original sources to present not only the basics and further developments of the established and, at the same time, subject to constant intervention in urology, but also the essential developments in the environment of neighboring medical disciplines, for example, think of laboratory medicine, radiology, nuclear medicine or rehabilitation medicine, but especially pathology. Incidentally, it was only these developments that created the basis for the correct setting of indications and the identification of alternatives to radical prostatovesiculectomy.

Die radikale Prostatovesikulektomie ist seit vielen Jahrzehnten die am häufigsten durchgeführte Therapieform des lokalisierten Prostatakarzinoms in Deutschland. Sie existierte bereits lange bevor diagnostische Methoden zur eindeutigen Identifizierung des Prostatakarzinoms beitragen konnten.

Freilich haben diese Entwicklungen auch zu einer besseren Abschätzbarkeit der Prognose des lokal begrenzten Prostatakarzinoms geführt, was naturgemäß weniger invasive Therapieformen förderte. So sank die Zahl der radikalen Prostatovesikulektomien zwischen 2008 und 2013 von über 30.000 auf gut 22.000 Operationen, insbesondere zugunsten der sog. aktiven Überwachung oder fokaler Therapien. Derweil scheint sich diese Entwicklung in den letzten Jahren wieder umzukehren mit knapp über 25.000 Eingriffen im Jahre 2018 [1]. Hierzu mag durchaus auch die gesteigerte Attraktivität der radikalen Prostatovesikulektomie durch das zwischenzeitlich nahezu flächendeckend verfügbare robotisch assistierte Verfahren beigetragen haben. So hat das Vorhandensein eines erfolgreichen radikalen Therapieverfahrens die Diagnostik des Prostatakarzinoms ebenso gefördert wie umgekehrt Fortschritte der Tumordiagnostik wiederum Auswirkungen auf die Therapiepräferenz zu haben scheinen.

## Anfänge der operativen Behandlung des Prostatakarzinoms

**Figure 1:**
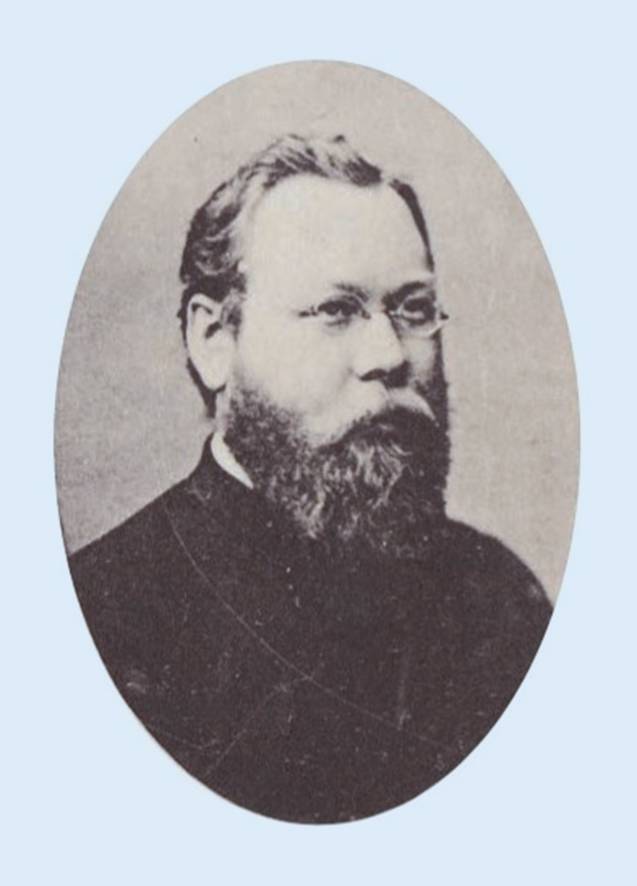

Seit der ersten radikalen Prostatovesikulektomie durch Hugh Hampton Young (1870–1945) im Juli 1904 sind mittlerweile fast 120 Jahre vergangen [2]. Dennoch ist die Prostatektomie, wenn auch inzwischen mit anderem Zugangsweg und zumeist mittels laparoskopischem Verfahren, nach wie vor die Standardtherapie bei der Behandlung des lokal begrenzten Prostatakarzinoms neben der perkutanen Strahlentherapie, die ebenfalls bereits seit 1910 Einzug in die Behandlung des Prostatakarzinoms fand [3]. Erste offenchirurgische Operationen, die die Entfernung der gesamten Prostata zum Ziel hatten, wurden aber bereits in den 1880er-Jahren beschrieben: etwa durch Heinrich Leisrink (1845–1885; Abb. 1). Der gebürtige Hamburger verließ seine Vaterstadt lediglich für das Studium der Medizin in Göttingen und Kiel und nochmals 1870, als er seine Stelle am Allgemeinen Krankenhaus kündigte, um im deutsch-französischen Krieg als Feldarzt dienen zu können. Später wirkte er bis zu seinem Tod mit 39 Jahren als Oberarzt der Chirurgie wesentlich an der Etablierung des Israelitischen Krankenhauses als Ort moderner operativer Praxis mit [4]. Im Jahre 1882 führte Leisrink dort die erste dokumentierte perineale Prostatektomie, noch unter Belassung der Samenblasen, durch [5]. Ähnliche Eingriffe wurden 1889 durch Belfield in Chicago und McGill in Leeds berichtet, die damit allerdings vor Etablierung endourologischer Möglichkeiten zur Desobstruktion der Prostata die benigne Prostatahyperplasie zu behandeln suchten [6, 7]. Einzelne weitere Fallbeschreibungen mit unterschiedlichen Operationsumfängen und -indikationen führten Hatzinger et al. in einer Veröffentlichung an [8].

## Etablierung der radikalen Prostatovesikulektomie

**Figure 2:**
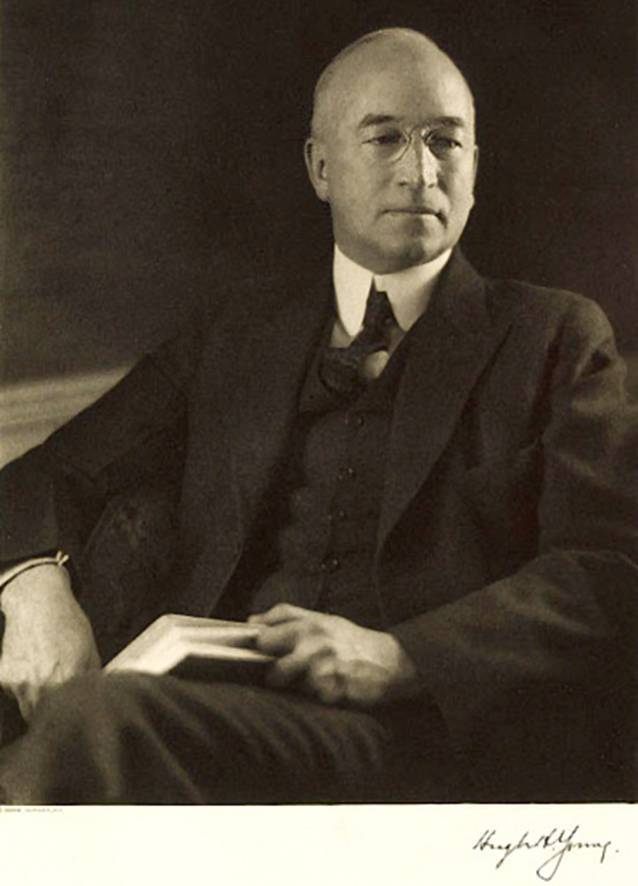

**Figure 3:**
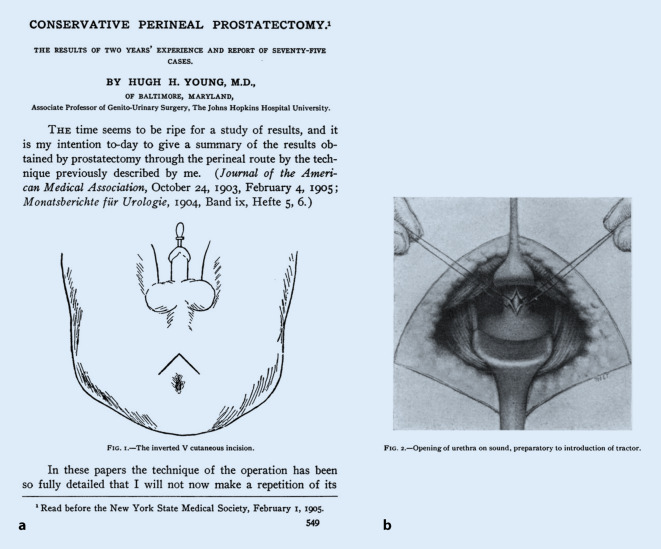

**Figure 4:**
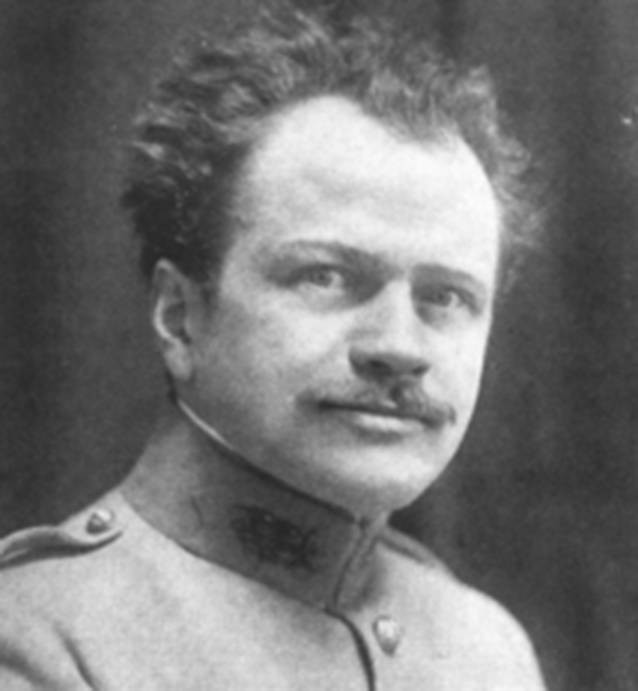

Zur Etablierung der onkologisch suffizienten operativen Behandlung des Prostatakarzinoms kam es erst einige Jahre später, wobei Hugh Hampton Young (1870–1945; Abb. 2) die erste solche Operation in vier Fällen vollumfänglich ausführen und beschreiben konnte [2]. Die Erfahrung Leisrinks, dass es dabei zu einer Verletzung des Rektums kommen kann, machten später auch Young und andere Operateure immer wieder, so dass Young bereits 1905 dokumentierte, lokal fortgeschrittene Tumoren einem solchen Eingriff besser nicht zuzuführen [2]. Der Meister gab in derselben Veröffentlichung aber auch Preis, wie sich rektourethrale Fisteln vermeiden ließen: „The suprapubic cystostomy adds nothing to gravity of the operation, and has changed the results obtained from constant failure to constant success“ [2]. Die Prostatektomie erfolgte damals und noch lange später in erster Linie von perineal (Abb. 3). Weitere Versuche wurden in der ersten Hälfte des 20. Jahrhunderts von ischiorektal, sakral und sakroperineal unternommen, durchgesetzt hat sich jedoch – auch gegenüber dem perinealen Zugang – der retropubische Ansatz [8]. Dieser wurde erstmals von René Leriche (1879–1955; Abb. 4), dem Chefarzt der Chirurgischen Klinik in Lyon, bereits im Jahre 1911 durchgeführt, trat seinen Siegeszug aber erst nach dem Zweiten Weltkrieg durch die Modifikationen von Terence J. Millin (1903–1980) an, der 1945 erstmals eine klassische retropubische Prostatektomie ohne die bei Leriche noch obligate Eröffnung der Harnblase durchführte [9]. Der Begriff *radikale Prostatektomie* wurde in der Literatur übrigens erstmals 1946 in einem Bericht von Ormond gewählt, wobei dieser wiederum von perineal operierte [10]. Erst 1954 berichteten Chute und McDonald unabhängig voneinander über eine *radikal retropubische Prostatektomie* [11, 12].

## Vor der Prostatektomie steht die Prostatabiopsie

Es ist heute selbstverständlich, dass der radikalen Prostatovesikulektomie eine histologische Diagnosesicherung vorangehen muss. Dies forderte im Übrigen schon Young, wobei erste Ansätze zur Aspirationsbiopsie zunächst von perineal durch Russel S. Ferguson 1933 unternommen wurden [13]. In der klinischen Routine setzte sich deutlich später, etwa ab Ende der 1960er-Jahre, eine modifizierte Feinnadelbiopsie der Prostata von transrektal durch. Diese war 1956 vom Zytologen Franzén in Stockholm am Karolinska-Institut entwickelt worden und griff dabei erste transrektale Biopsieansätze von Astraldi in der vorantibiotischen Zeit auf [14]. Diese Untersuchungen fanden bis weit in die 1980er-Jahre fingergeführt statt, wobei palpatorisch auffällige Areale 2‑mal punktiert wurden. Eine erste Veröffentlichung zur Sonographie gesteuerten Biopsie der Prostata, zunächst von perineal, aber unter transrektaler Ultraschallkontrolle, erschien im Jahre 1983 [15, 16]. Lee et al. machten 1985 publik, dass die transrektale Ultraschalluntersuchung ein sehr geeignetes Mittel zur Identifikation suspekter Areale sei [17]. Hodge et al. von der Stanford University unternahmen 1989 eine erste Untersuchung unter Anwendung der randomisierten transrektalen Sextantenbiopsie, die sich in den darauf folgenden Jahren als diagnostisches Mittel der Wahl durchsetzen konnte [18]. Seit Anfang der 2000er-Jahre kam es zum steten Versuch, Diagnosesicherheit bei überschaubaren Risiken zu optimieren. Durchgesetzt hat sich in den letzten Jahren die 12-fach-Stanzbiopsie der Prostata [19, 20]. Weitere Optimierungen der Sensitivität dieses für die Diagnostik des Prostatakarzinoms so wichtigen Verfahrens folgten: bereits 1995 veröffentlichte eine Arbeitsgruppe aus den USA erste Daten zur Elastographie, die sich unterschiedlichen Dichtewerten von benignem und malignem Gewebe der Prostata widmet [21]. Gleichwohl fand dieses diagnostische Mittel nie flächendeckend Eingang in die Routinediagnostik. Im Gegensatz dazu konnte die kernspintomographische Unterstützung der randomisierten Prostatabiopsie in den letzten Jahren die Diagnostik des Prostatakarzinoms optimieren. Nach ersten Untersuchungen ab 2010 gilt die MRT-gestützte Fusionsbiopsie der Prostata heute als Mittel der Wahl zumindest bei allen Patienten, bei denen nach benignem Biopsieergebnis weiter Tumorverdacht besteht [22, 23].

## Vom Rollenwechsel des Prostatakarzinoms in der Pathologie des 20. Jahrhunderts

**Figure 5:**
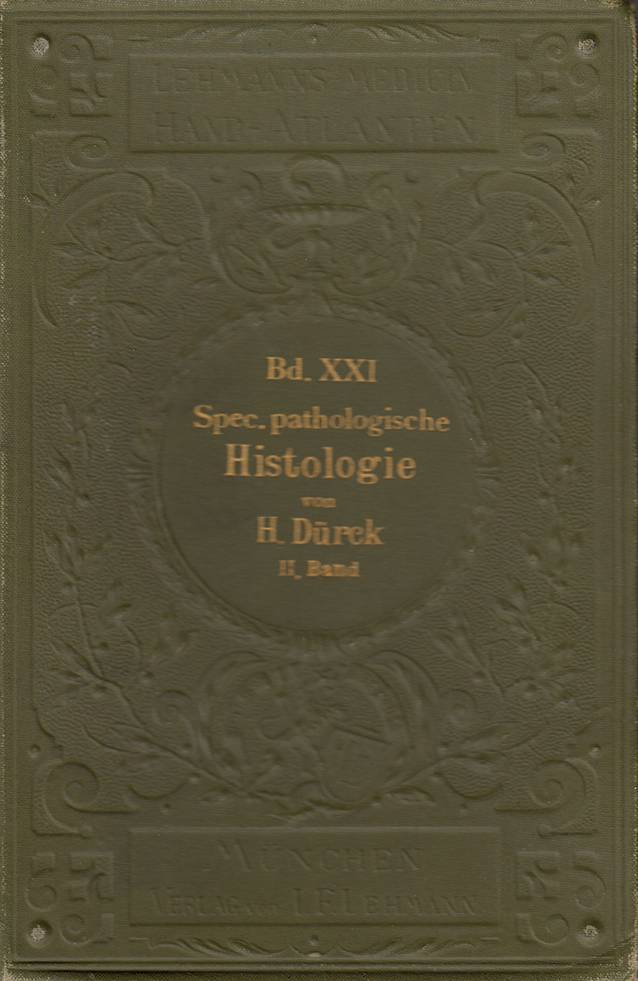

Doch zurück in die frühen Jahre der Prostatachirurgie und noch etwas weiter. Ist das Prostatakarzinom heute die häufigste zu diagnostizierende Malignomerkrankung des Mannes, so ging man noch Mitte des 19. Jahrhunderts davon aus, dass Karzinome der Prostata eine eher seltene Tumorentität darstellen [24]. Offensichtlich gab es v. a. deutlich weniger Möglichkeiten, diese Tumoren – bei einem freilich auch deutlich geringeren Bevölkerungsanteil von Männern im entsprechend gefährdeten Alter als heute – zu detektieren. Entsprechend schwierig scheint sich lange auch die histologische Sicherung dargestellt haben. Heute ist die Uropathologie eine integrale und keineswegs die unbedeutendste Disziplin der pathologischen Wissenschaft und Praxis. Das war insbesondere, was die Prostata betrifft, in den Pionierjahren keineswegs der Fall. Es ist angesichts der oben beschriebenen frühen Geschichte der Prostatektomie nicht verwunderlich, aber doch bezeichnend, dass ein im Jahre 1901 erschienenes Standardwerk zur *Speciellen pathologischen Histologie* (Abb. 5 und 6) maligne Erkrankungen der Prostata oder gar die Bezeichnung Prostatakarzinom selbst nicht nennt. Auf gerade einmal zwei Druckseiten beschäftigte sich Herausgeber Hermann Ludwig Dürck (1869–1941), der Schüler Otto von Bollingers (1843–1909) und Hans Chiaris (1851–1916) war, damals mit der Prostata an sich, während – den Vorsprung der Nierenchirurgie in dieser Zeit gut abbildend – gut- und bösartigen Erkrankungen der Niere über 50 Seiten gewidmet waren. Neben Fibromen, Lipomen, Leiomyo- und Rhabdosarkomen war Dürck auch schon der Name Paul Grawitz (1850–1932) als Eponym bekannt, der „zuerst richtig erkannte, […] dass die häufigsten epithelialen Neubildungen in der Niere von verlagerten Nebennierenkeimen abstammten“ [25]. Zum Prostatakarzinom wie gesagt dagegen kein Wort des damaligen Privatdozenten der Universität München, der später Ordinarius für Pathologie in Jena war und ab 1911 als Direktor des Pathologischen Institut des damals noch nicht universitären Klinikums rechts der Isar in seiner Heimatstadt München fungierte [26].

**Figure 6:**
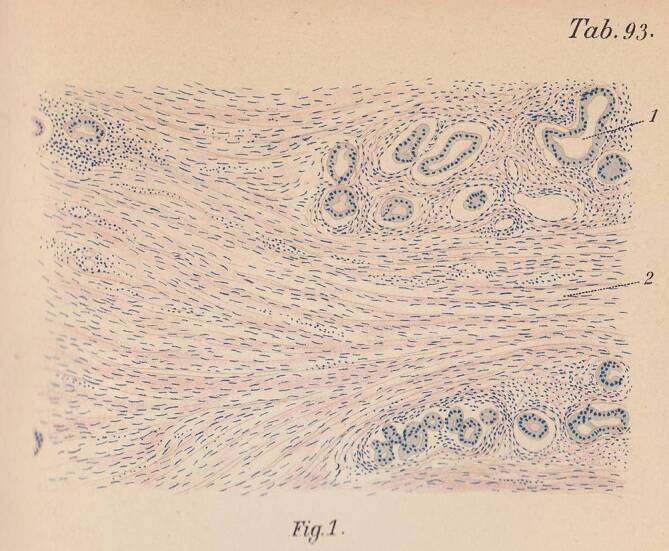

## Donald F. Gleason als Wegbereiter moderner Prostatapathologie

**Figure 7:**
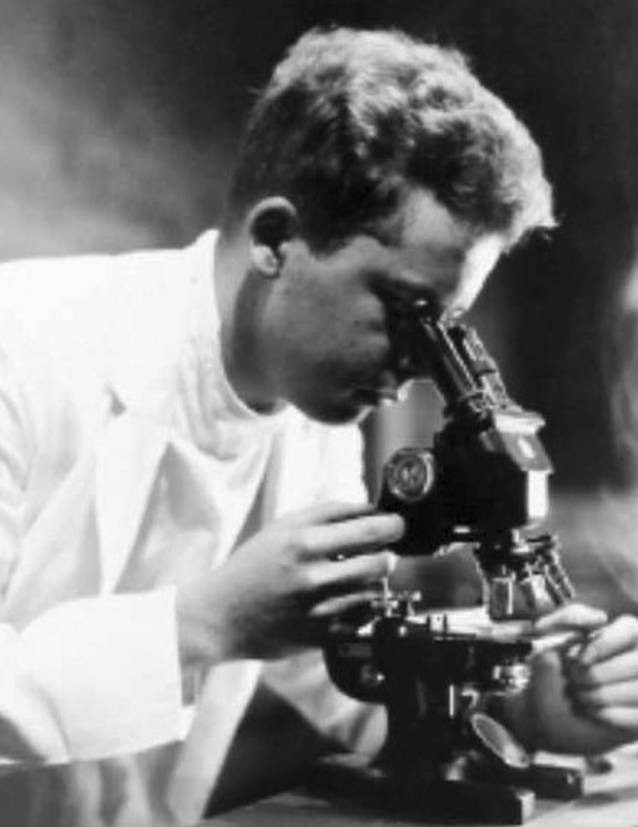

Das Prostatakarzinom wurde erst von den folgenden Pathologengenerationen „entdeckt“, wobei hierfür wie Jahrzehnte zuvor bei der Niere die Fortschritte der Urologen, die sich damals in der Hauptsache wohl eher als *specielle Urochirurgen* verstanden wissen wollten, bei der operativen Therapie des Prostatakarzinoms wesentlich verantwortlich waren [27]. Und wiederum zusammenhängend damit die sich ab den 1960er-Jahren durchsetzende präoperative Biopsie der Prostata, die der Pathologie eine ganz zentrale Bedeutung für die Diagnosestellung des Prostatakarzinoms einräumte. Zunächst wurden die Aspirationsbiopsien zytologisch untersucht, der Erstbeschreiber dieses Verfahrens, Franzén, war schließlich Zytologe gewesen [14]. Feinnadelpunktionen mit der TRU-cut-Methode ermöglichten bald auch Asservierung zusammenhängender Gewebeproben [15]. Diese beiden Formen der Diagnosestellung koexistierten für etwa 20 Jahre. Erst der Siegeszug des Gleason-Gradings als diagnostische, aber auch prognostische Kategorisierung des Prostatakarzinoms führte zur Durchsetzung der Feinnadelbiopsie. Ab 1960 arbeitete der Pathologe Donald F. Gleason (1920–2008) (Abb. 7) im Rahmen der Veteran’s Administration Cooperative Urology Research Group (VACURG) auf Initiative des Urologen George Mellinger an einem System zur histologischen Beurteilung von Prostatakarzinomgewebe [28]. Das Gradingverfahren nach Gleason, das sich im Laufe der 1980er-Jahre als prognostisch signifikantes System durchgesetzt hat, wurde erstmals 1966 in einer kurzen Monographie beschrieben und 1974 in ihrer heutigen Form als Gradingsystem veröffentlicht, nachdem der Erstbeschreiber etwa 4000 Fälle beurteilt hatte [28–30]. Relativ früh wurden aber auch andere Prognosekriterien des Prostatakarzinoms beschrieben, etwa schon 1967 durch Pennington et al., die die Bedeutung der perineuralen Lymphinvasion im Prostatektomiepräparat identifiziert hatten [31].

## Bildgebung: der Siegeszug nuklearmedizinischer Verfahren

Es ist sattsam bekannt, dass die Prostatastanzbiopsie erst in den letzten drei Jahrzehnten durch die Zunahme der PSA-basierten Vorsorge ihren heutigen Stellenwert erreicht hat. Eine andere – in größter Wertschätzung – zu nennende medizinische Auxiliardisziplin hatte noch vor der Pathologie oder Labormedizin für die das Prostatakarzinom behandelnden Urologen eine weit größere Bedeutung. Denn die Zeiten, als das Prostatakarzinom nicht inzidentell oder im Rahmen der Vorsorge festgestellt wurde, sondern in der Mehrheit als bereits ossär metastasierter Tumor, liegen noch nicht einmal eine Medizinergeneration zurück. Hier kam die Radiologie und ihr einstiges Teilgebiet Nuklearmedizin ins Spiel. Ehe ab 1952 erste experimentelle Untersuchungen zur Etablierung der Knochenszintigraphie begannen, war die Diagnostik auf ossäre Metastasen rein radiologisch verfasst [33]. Es dauerte sogar noch bis Mitte der 1960er-Jahre, ehe eine erste Veröffentlichung zur Skelettszintigraphie beim Prostatakarzinom publiziert werden konnte [34]. Schon früh war die Überlegenheit des neuen Verfahrens – unabhängig von der Art des verwendeten Radionuklids – gegenüber der konventionellen Röntgenuntersuchung offensichtlich: mit den technischen Möglichkeiten der 1970er-Jahre waren im Röntgenbild ossäre Destruktionen erst aber einer Zerstörung von Wirbelkörpern um 40–50 % sichtbar, weit weniger fortgeschrittene Störungen bereits mit der Szintigraphie [35]. Zunächst wurde dazu ^18^Fluor verwendet, 1971 berichtete die Baseler Radiologie in Kooperation mit dem urologischen Ordinarius Georg Rutishauser erstmals über eine ^85^Strontium-Szintigraphie [36]. Zwei Jahre später folgte zum ersten Mal die Anwendung des weit weniger strahlenbelastenden ^99m^Technetiums, das bis heute das Radionuklid der Wahl darstellt [37].

**Figure 8:**
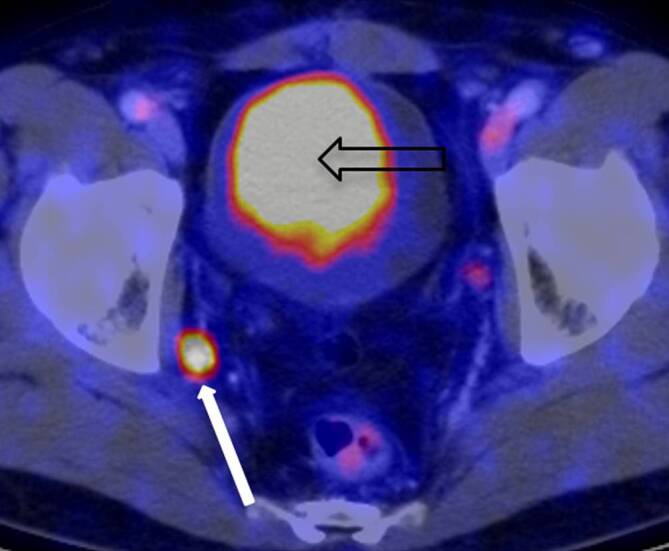

Durch einen als revolutionär zu bezeichnenden Wandel im Auftreten des Prostatakarzinoms hat sich der Stellenwert der Knochenszintigraphie freilich zwischenzeitlich gewandelt von einem Instrument der Erstdiagnose von zuvor klinisch inapparenten Tumoren hin zu einem Verlaufsdiagnostikum insbesondere zur Therapiekontrolle, sie ist und bleibt aber unverzichtbares Hilfsinstrument der uroonkologischen Therapie. So wie die Skelettszintigraphie ab den 1970er-Jahren mehr und mehr die konventionelle Röntgendiagnostik als Diagnostikum der Wahl von ossären Filiae ablöste, ist in den letzten Jahren ein weiterer Erfolg der Nuklearmedizin über die Radiologie zu beobachten: war die Computertomographie des Abdomens, erstmals 1977 im Zusammenhang mit dem Prostatakarzinom in Erscheinung getreten, ab den 1980er-Jahren ein hochwillkommener neuer Routineansatz zur Detektion von viszeralen Metastasen, so wird dieses Verfahren seit gut 10 Jahren – zumindest dort, wo entsprechende Einrichtungen vorgehalten werden – erneut nuklearmedizinisch durch die Etablierung des ^68^Gallium-PSMA-PET-CT (Abb. 8) bei der Detektion von kleinsten lymphogenen und viszeralen Metastasen ersetzt oder zumindest ergänzt [38, 39]. Die Radiologie ist gleichwohl auch heute noch von großer Bedeutung für die Prostatakarzinomdiagnostik. Wie bereits weiter oben angedeutet seit knapp 10 Jahren zunehmend in Form der Magnetresonanztomographie der Prostata im Rahmen der Primärdiagnostik vor bzw. zur Unterstützung einer gezielten Prostatastanzbiopsie [40].

## Revolution aus dem Labor: Entwicklung und Bedeutung des prostataspezifischen Antigens (PSA)

Dass die Häufigkeit der früher bei Diagnosestellung dominierenden, lokal fortgeschrittenen oder bereits metastasierten Prostatakarzinome heute massiv zurückgegangen ist, dürfte ganz wesentlich mit der Entdeckung, Weiterentwicklung und klinische Nutzbarmachung des PSA zwischen 1970 und 1980 mit den grundlegenden Arbeiten von Abling et al., Wang et al. und Papsidero et al. in Zusammenhang stehen [41–43]. Egal, ob nun durch zytologische oder histopathologische Auswertung von durch Biopsie gewonnene Gewebeproben: bis weit in die 1980er-Jahre hinein war nur der klinische Hinweis auf ein Prostatakarzinom die Indikation für eine Biopsie der Prostata, entweder durch suspekten Tastbefund, Symptome des lokal fortgeschrittenen Primärtumors oder Nachweis von Knochenmetastasen. Eine Untersuchung von Desireddi et al. an über 3400 Patienten, die von einem Urologen in Chicago (USA) prostatektomiert wurden, zeigte einen klaren Vorteil im progressionsfreien Überleben für jene Patientengruppe, die in der PSA-Ära (dort ab 1992) behandelt wurden gegenüber den zuvor diagnostizierten Patienten [44]. Obwohl in diversen Studien gezeigt werden konnte, dass in Kollektiven, die sich einer regelmäßigen PSA-basierten Vorsorge unterziehen, mehr lokal begrenzte und damit besser behandelbare Prostatakarzinome und weniger fortgeschrittene Tumorstadien festgestellt werden als in PSA-naiven Personengruppen, so ist das sog. PSA-Screening nach wie vor umstritten. Das liegt v. a. an der Sorge vor Überdiagnostik und -therapie mit der damit verbundenen Folge (vermeidbarer) Komplikationen wie postoperativer Belastungsharninkontinenz und erektiler Dysfunktion [45].

## Als die Rehabilitationsmedizin unverzichtbarer Bestandteil der Prostatakarzinomtherapie wurde

Mit den funktionellen Folgen der Prostatektomie beschäftigte sich erstmals 1945 eine Veröffentlichung von Goldstein und Rubin, die sich mit der postoperativen Inkontinenz befasste [46]. Schnell wurde klar, dass die Stärkung der Beckenbodenmuskulatur die Rekonvaleszenz fördert, bald experimentierte man mit der Implantation elektrischer Impulsgeber, ein Verfahren, das sich nicht durchsetzen konnte [47, 48]. Als externe Elektrotherapie hat die Grundannahme insbesondere im Rahmen eines Biofeedback-Ansatzes ab Mitte der 1970er-Jahre jedoch bis in unsere Zeit überdauert [49]. Diese Behandlungen müssen rasch nach der Entfernung des transurethralen Katheters gestartet werden, man geht heute sogar dazu über, Patienten bereits vor der radikalen Prostatektomie mit den Beckenboden stärkenden Übungen beginnen zu lassen. In zahlreichen Rehabilitationsmedizinischen Einrichtungen werden solche Maßnahmen als ein wesentlicher Bestandteil der Rekonvaleszenz in zumeist 3‑wöchigen stationären Aufenthalten trainiert. Gleichwohl ist nicht jedes Ausmaß einer postoperativen Belastungsharninkontinenz auf diese Weise zufriedenstellend korrigierbar. Schon 1983 berichtete man daher vom Einsatz eines artifiziellen Schließmuskels, dessen Weiterentwicklung AS800 rasch zur Standardtherapie bei ausgeprägter Inkontinenz wurde [50, 51]. Nach dem Jahr 2000 konnten sich allmählich weniger komplikationsträchtige Schlingenverfahren nach dem Vorbild entsprechender Versorgung weiblicher Belastungsharninkontinenz durchsetzen, die heute bei weniger ausgeprägter postoperativer Inkontinenz zum Einsatz kommen [52].

Lange Zeit wenig beachtet wurde eine weitere häufige Folgeerscheinung der radikalen Prostatektomie: erste Veröffentlichungen zur postoperativen erektilen Dysfunktion gehen zumeist auf Finkle et al. zurück, die ab 1960 zu dieser Thematik publizierten [53]. Diese Arbeitsgruppe beschrieb 1981 in einem zugegebenermaßen relativ kleinen Kollektiv eine „normale“ Potenz in 43 % der operierten Patienten [54]. Ein Jahr später führte Patrick Walsh am 26. April 1982 die erste nervschonende radikale Prostatektomie durch, ein Verfahren, das bereits unter den ersten Patienten in der Folge eine zufriedenstellende erektile Funktion bei 86 % der Patienten ein Jahr postoperativ zur Folge hatte [55]. Außer dieses bereits bei Therapie die anatomischen Strukturen der erektilen Potenz schonenden Operationsverfahrens hatte man lange jedoch wenig Möglichkeiten zur Verbesserung der Situation. Die intrakavernöse Injektion von Papaverin, die im Laufe der 1980er-Jahre in die SKAT-Therapie mündete, war lange Zeit die einzige, wenn auch durchaus nicht Komplikationen freie Behandlungsstrategie [56]. Bereits 1982 wurde in den USA die Vakuumpumpe nach Osborn zur nicht-medikamentösen Wiedererlangung der Potenz eingeführt [57]. Einen revolutionären Effekt auch für die Wiedererlangung der postoperativen Potenz hatte 1998 die Zulassung des Phosphodiesterasehemmers Sildenafil, der heute neben anderen PDE-5-Hemmern nicht nur für die Bedarfs- sondern auch als niedrigdosierte Dauertherapie zur verbesserten Schwellkörperdurchblutung eingesetzt wird [58].

## Einzug von „high technology“ in die Urologie: über die laparoskopische Lymphadenektomie zur robotisch assistierten Prostatektomie

**Figure 9:**
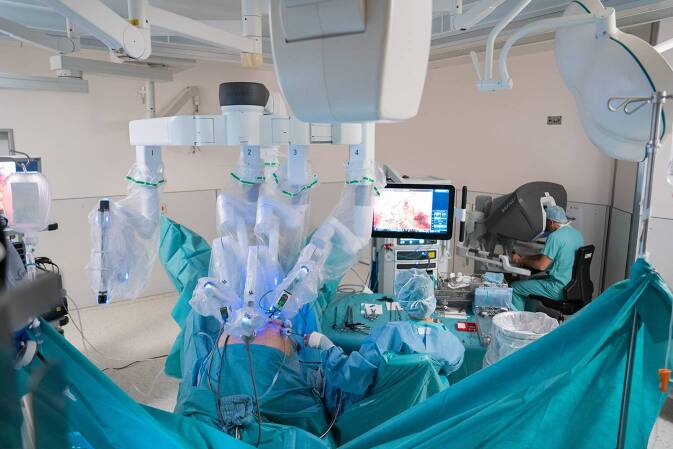

Nach diesem Ausflug zu diversen Umfelddisziplinen und ihrem Beitrag zu Diagnostik und Therapie des Prostatakarzinoms gebührt der Schlusspunkt den Neuerungen auf dem Gebiet der Urologie selbst. Etwa zeitgleich mit der Verfügbarkeit des PDE-5-Hemmers Sildenafil betrat im Jahr 1997 die laparoskopische radikale Prostatektomie die Bühne der medizinischen Weltliteratur mit einer Arbeit von Schuessler et al., die Erfahrungen der ersten neun in den USA mit dieser Methode operierten Patienten dokumentierten [59]. Hervorgegangen war dieser neue Ansatz aus der sich Anfang der 1990er-Jahre etablierenden laparoskopischen (Staging‑)Lymphadenektomie, die bis zum Beginn des neuen Jahrhunderts der retroperitonealen radikalen Prostatektomie stets vorgeschaltet war. Die laparoskopische Lymphknotendissektion wurde 1990 parallel in Los Angeles, Charlston (USA), Saragossa (ES) und Heidelberg (D) erstmals durchgeführt [60–63]. Zeitgleich zur schrittweisen Weiterentwicklung der laparoskopischen Lymphadenektomie zur endoskopischen extraperitonealen radikalen Prostatektomie (EERPE) durch Renaud Bollens, Jens Rassweiler und Jens-Uwe Stolzenburg kam es in Mitteleuropa und den USA auch zur ersten robotisch assistierten laparoskopischen Prostatektomie (RALP) durch Mani Menon [64–67]. Im Jahre 1999 war das daVinci Surgical-System (Intuitive Surgery Inc, Sunnyvale, CA, USA; Abb. 9) auf den medizinischen Markt gekommen, um die koronare Bypassoperation zu erleichtern, schon im Jahre 2000 konnten Menon in den Vereinigten Staaten und Binder et al. in Frankfurt/Main (D) das Verfahren erstmals beim Prostatakarzinom anwenden und publizieren [68, 69].

Technische Verbesserungen sowohl der apparativen Voraussetzungen als auch intraoperativer Verfahrensschritte haben sich analog zur Entwicklung bei der offenchirurgischen Prostatektomie, nur sehr viel rascher, auch bei der robotisch assistierten laparoskopischen Prostatektomie ergeben [69]. Bereits 2004 stellten etwa Su et al. eine erste Kohorte von Patienten vor, bei denen ein nervschonendes Verfahren Anwendung fand [70]. Einigen Patienten heute besonders wichtige Aspekte und Wünsche – schnellere Rekonvaleszenz, kürzerer Krankenhausaufenthalt, reduzierter Schmerzmittelgebrauch – werden von der robotisch assistierten radikalen Prostatektomie fraglos erfüllt [71–73]. Um Vorteile im funktionellen Langzeitoutcome als gesichert anzuerkennen, dazu fehlen heute jedoch eindeutige Ergebnisse [74–76]. Resultate, die für das progressionsfreie Überleben der Patienten angesichts des bereits höchst ausgefeilten offenchirurgischen Verfahrens freilich wohl gar nicht erst erwartet werden durften.

Dennoch hat sich, trotz deutlich höherer Therapiekosten, das robotisch assistierte Verfahren der radikalen Prostatektomie 20 Jahre nach seiner Erstbeschreibung nicht nur in den USA, sondern auch in weiten Teilen Europas, insbesondere in Deutschland, heute durchgesetzt. Inwieweit dies angesichts hoher Anschaffungs- und Wartungskosten flächendeckend so bleiben wird, könnte am Ende weniger von der dem Fach Urologie schon immer innewohnenden Innovationsfreudigkeit, sondern mehr vom tatsächlichen Umfang der heraufziehenden Krise des Wirtschafts- und Gesundheitswesens infolge der COVID-19-Pandemie abhängen. Gut möglich, dass die hierzulande in den letzten Jahren wieder zugenommene Anzahl von radikalen Prostatovesikulektomien, die am ehesten auf die gestiegene Attraktivität des minimal-invasiven Ansatzes zurückzuführen sein könnte, bei Low-risk-Tumoren während der Pandemie wieder zugunsten der aktiven Überwachung abnimmt. Dies wird aber erst die Zukunft zeigen.

## Fazit für die Praxis

- Die radikale Prostatektomie mit Ursprung in den 1880er-Jahren und Etablierung zu Beginn des 20. Jahrhunderts, war – vom Wandel des Zugangsweges abgesehen – über die Jahrzehnte kaum wesentlichen Veränderungen unterworfen.
- Entwicklungen zur Diagnostik des Prostatakarzinoms kamen erst nach und nach, wohl aber auch durch das Vorhandensein einer etablierten Therapiemethode, in Gang.
- Namentlich sind hier der radiologische, später auch szintigraphische Nachweis von Knochenmetastasen, vor allem aber – was lokale Tumoren betrifft – die Entdeckung und klinische Einführung des Prostata spezifischen Antigens (PSA) in den 1970er- und 1980er-Jahren zu nennen.
- Insbesondere die Etablierung der bioptischen Diagnosesicherung, eng verknüpft mit der Einführung der pathologischen Klassifizierung nach Gleason, markierte parallel zur flächendeckenden Anwendung der PSA-Messung einen Meilenstein in der Diagnostik von Prostatakarzinomen.
- Mit der zunehmenden Etablierung minimal-invasiver Verfahren wie laparoskopischer oder robotisch assistierter Prostatektomie scheint der urologische Leiteingriff wieder an Attraktivität gegenüber weniger invasiven Eingriffen gewonnen zu haben.

## References

[1] Statistisches Bundesamt Deutschland (2018) Operationen und Prozeduren an vollstationären Patienten: Bundesländer, Jahre, Operationen und Prozeduren. https://www-genesis.destatis.de/genesis/online?operation=ergebnistabelleUmfang&levelindex=2&levelid=1598372474037&downloadname=23141-0110#abreadcrumb. Zugegriffen: 24. Aug. 2020

[2] Young HH (1905). VIII. Conservative perineal prostatectomy: the results of two years’ experience and report of seventy-five cases. Ann Surg.

[3] Bagshaw MA, Kaplan ID, Cox RC (1993). Prostate cancer. Radiation therapy for localized disease. Cancer.

[4] Jenss H (2016). Der Chirurg Heinrich Leisrink und seine Bedeutung für das Israelitische Krankenhaus. Israelitisches Krankenhaus in Hamburg – 175 Jahre.

[5] Leisrink H, Ahlsberg A (1882). Tumor prostatae: Totale Exstirpation der Prostata. Arch Klin Chir.

[6] NN (1889) Suprapubic Prostatectomy. Ind Med Gaz 24(11):342–343. PMC5141855 29000254

[7] Raymond G, Chevallier D, Amiel J (1988). 1987: the 100th anniversary of transvesical prostatic adenomectomy. J Urol.

[8] Hatzinger M, Hubmann R, Moll F, Sohn M (2012). Die Geschichte der Prostatektomie – Von den Anfängen bis DaVinci. Aktuelle Urol.

[9] Millin T (1945). Retropubic prostatectomy; a new extravesical technique; report of 20 cases. Lancet.

[10] Ormond JK (1946). Radical perineal prostatectomy for carcinoma of prostate. Surgery.

[11] Chute R (1954). Radical retropubic prostatectomy for cancer. J Urol.

[12] McDonald HP, Upchurch WE, Sturdevant CE (1954). Perineal biopsy combined with radical retropubic prostatectomy for early carcinoma of the prostate.

[13] Ferguson RS (1933). Recent advances in the diagnosis of carcinoma of the prostate. Can Med Assoc J.

[14] Franzen S, Giertz G, Zajicek J (1960). Cytological diagnosis of prostatic tumours by transrectal aspiration biopsy: a preliminary report. Br J Urol.

[15] Chiari R, Harzmann R (1975). Perineale und transrektale Stanzbiopsie der Prostata. Urologe A.

[16] Rifkin MD, Kurtz AB, Goldberg BB (1983). Prostate biopsy utilizing transrectal ultrasound guidance: diagnosis of nonpalpable cancers. J Ultrasound Med.

[17] Lee F, Gray JM, McLeary RD, Meadows TR, Kumasaka GH, Borlaza GS, Straub WH, Lee F, Solomon MH, McHugh TA (1985). Transrectal ultrasound in the diagnosis of prostate cancer: location, echogenicity, histopathology, and staging. Prostate.

[18] Hodge KK, McNeal JE, Terris MK, Stamey TA (1989). Random systematic versus directed ultrasound guided transrectal core biopsies of the prostate. J Urol.

[19] Naughton CK, Miller DC, Mager DE, Ornstein DK, Catalona WJ (2000). A prospective randomized trial comparing 6 versus 12 prostate biopsy cores: impact on cancer detection. J Urol.

[20] Fink KG, Hutarew G, Pytel A, Esterbauer B, Jungwirth A, Dietze O, Schmeller NT (2003). One 10-core prostate biopsy is superior to two sets of sextant prostate biopsies. BJU Int.

[21] Rubens DJ, Hadley MA, Alam SK, Gao L, Mayer RD, Parker KJ (1985). Sonoelasticity imaging of prostate cancer: in vitro results. Radiology.

[22] Turkbey B, Xu S, Kruecker J, Locklin J, Pang Y, Shah V, Bernardo M, Baccala A, Rastinehad A, Benjamin C, Merino MJ, Wood BJ, Choyke PL, Pinto PA (2011). Documenting the location of systematic transrectal ultrasound-guided prostate biopsies: correlation with multi-parametric MRI. Cancer Imaging.

[23] Heidenreich A, Bastian PJ, Bellmunt J, Bolla M, Joniau S, van der Kwast T, Mason M, Matveev V, Wiegel T, Zattoni F, Mottet N, European Association of Urology (2014). EAU guidelines on prostate cancer. part 1: screening, diagnosis, and local treatment with curative intent-update 2013. Eur Urol.

[24] Adams J (1851). The Anatomy and Diseases of the Prostate Gland.

[25] Dürck H, Dürck H (1901). Harnorgane. Atlas und Grundriss der speciellen pathologischen Histologie.

[26] Schnalke T (1991). Hermann Dürck (1869–1941). Pathologe.

[27] Young RH, Eble JN (2019). The history of urologic pathology: an overview. Histopathology.

[28] Phillips JL, Sinha AA (2009). Patterns, art, and context: Donald Floyd Gleason and the development of the Gleason grading system. Urology.

[29] Gleason DF (1966). Classification of prostatic carcinomas. Cancer Chemother Rep.

[30] Gleason DF, Mellinger GT (1974). Prediction of prognosis for prostatic adenocarcinoma by combined histological grading and clinical staging. J Urol.

[31] Pennington JW, Prentiss RJ, Howe G (1967). Radical prostatectomy for cancer: significance of perineural lymphatic invasion. J Urol.

[32] Łukasz Nyk et al (2016) Postępy Nauk Medycznych 11:841–845

[33] Riccabona G (1999). Nuclear medicine in diagnosis and therapy of bone and joint diseases. Nucl Med Rev Cent East Eur.

[34] Simon N, Feitelberg S, Glickman SI (1967). Scanning radioactive phosphorus in bone metastases from cancer of the prostate. J Urol.

[35] Ell PJ (1975). The clinical role of skeletal scanning. Ann R Coll Surg Engl.

[36] Graber P, Rutishauser G, Baumann JM (1971). Eine vergleichende Bewertung von saurer Serumphosphatase, konventioneller Röntgendiagnostik, Knochenbiopsie und Sr-85-Szintigraphie als Methoden zum Nachweis von Knochenmetastasen beim Prostatacarcinom. Urologe.

[37] Redman JF, Turley JT (1973). Technetium polyphosphate bone scans in carcinoma of prostate. Urology.

[38] Jentsch F, Stringaris K, Kaiser G, Kirschner H (1977). Computertomographie des kleinen Beckens als Grundlage für strahlentherapeutisches Vorgehen, Lokalisation und Bestrahlungsplanung beim Prostata-Carcinom. Radiologe.

[39] Maurer T, Eiber M, Fanti S, Budäus L, Panebianco V (2016). Imaging for prostate cancer recurrence. Eur Urol Focus.

[40] Kayano PP, Carneiro A, Castilho TML, Sivaraman A, Claros OR, Baroni RH, Garcia RG, Mariotti GC, Smaletz O, Filippi RZ, Lemos GC (2018). Comparison of Gleason upgrading rates in transrectal ultrasound systematic random biopsies versus US-MRI fusion biopsies for prostate cancer. Int Braz J Urol.

[41] Ablin RJ, Bronson P, Soanes WA, Witebsky E (1970). Tissue- and species-specific antigens of normal human prostatic tissue. J Immunol.

[42] Wang MC, Valenzuela LA, Murphy GP, Chu TM (1979). Purification of a human prostate specific antigen. Invest Urol.

[43] Papsidero LD, Wang MC, Valenzuela LA, Murphy GP, Chu TM (1980). A prostate antigen in sera of prostatic cancer patients. Cancer Res.

[44] Desireddi NV, Roehl KA, Loeb S, Yu X, Griffin CR, Kundu SK, Han M, Catalona WJ (2007). Improved stage and grade-specific progression-free survival rates after radical prostatectomy in the PSA era. Urology.

[45] Ilic D, Djulbegovic M, Jung JH, Hwang EC, Zhou Q, Cleves A, Agoritsas T, Dahm P (2018). Prostate cancer screening with prostate-specific antigen (PSA) test: a systematic review and meta-analysis. BMJ.

[46] Goldstein AE, Rubin SW (1946). Urinary incontinence following suprapubic prostatectomy; with report of three cases. Urol Cutaneous Rev.

[47] Hutch JA, Fisher R (1968). Continence after radical prostatectomy. Br J Urol.

[48] Caldwell KP, Cook PJ, Flack FC, James D (1968). Treatment of post-prostatectomy incontinence by electronic implant. Br J Urol.

[49] Sotiropoulos A, Yeaw S, Lattimer JK (1976). Management of urinary incontinence with electronic stimulation: observations and results. J Urol.

[50] Barrett DM, Furlow WL (1983). Radical prostatectomy incontinence and the AS791 artificial urinary sphincter. J Urol.

[51] Bitker MO, Barrou B, Aranda B, Jardin A, Perrigot M, Chatelain C (1987). Results of treatment of postoperative urinary incontinence in men by implantation of an AS 800 artificial sphincter. Apropos of 15 cases. J Urol.

[52] Comiter CV (2002). The male sling for stress urinary incontinence: a prospective study. J Urol.

[53] Finkle AL, Saunders JB (1960). Sexual potency in aging males. III. Technic of avoiding nerve injury in perineal prostatic operations. Am J Surg.

[54] Finkle AL, Taylor SP (1981). Sexual potency after radical prostatectomy. J Urol.

[55] Walsh PC, Mostwin JL (1984). Radical prostatectomy and cystoprostatectomy with preservation of potency. Results using a new nerve-sparing technique. Br J Urol.

[56] Papadopoulos I, Weichert-Jacobsen K, Wand H (1990). Priapismus als Notfall-Folge der intrakavernösen Injektion vasoaktiver Substanzen. Z Urol Nephrol.

[57] Brison D, Seftel A, Sadeghi-Nejad H (2013). The resurgence of the vacuum erection device (VED) for treatment of erectile dysfunction. J Sex Med.

[58] Osmonov DK, Jünemann KP, Bannowsky A (2017). The “Kiel concept” of long-term administration of daily low-dose Sildenafil initiated in the immediate post-prostatectomy period: evaluation and comparison with the international literature on penile rehabilitation. Sex Med Rev.

[59] Schuessler W, Schulam P, Clayman R (1997). Laparoscopic radical prostatectomy: initial short-term experience. Urology.

[60] Gershman A, Daykhovsky L, Chandra M, Danoff D, Grundfest WS (1990). Laparoscopic pelvic lymphadenectomy. J Laparoendosc Surg.

[61] Rioja Sanz C, Mínguez Pemán JM, Blas MM, Martínez Bengoechea J, Rioja Sanz LA (1991). Laparoscopic lymphadenectomy for lymphatic staging in prostatic cancer: preliminary experience. Actas Urol Esp.

[62] Tierney JP, Kusminsky RE, Boland JP, Oliver RS (1991). Laparoscopic pelvic lymph node dissection. W V Med J.

[63] Beer M, Staehler G, Dörsam J (1991). Pelvine laparoskopische Lymphadenektomie. Indikationen, Technik, erste Ergebnisse. Chirurg.

[64] Bollens R, Vanden Bossche M, Roumeguere T, Damoun A, Ekane S, Hoffmann P, Zlotta AR, Schulman CC (2001). Extraperitoneal laparoscopic radical prostatectomy. Results after 50 cases. Eur Urol.

[65] Rassweiler J, Frede T, Seemann O, Stock C, Sentker L (2001). Telesurgical laparoscopic radical prostatectomy. Initial experience. Eur Urol.

[66] Stolzenburg JU, Do M, Pfeiffer H, König F, Aedtner B, Dorschner W (2002). The endoscopic extraperitoneal radical prostatectomy (EERPE): technique and initial experience. World J Urol.

[67] Jeong W, Kumar R, Menon M (2016). Past, present and future of urological robotic surgery. Investig Clin Urol.

[68] Binder J, Kramer W (2001). Robotically-assisted laparoscopic prostatectomy. BJU Int.

[69] Turpen R, Atalah H, Su LM (2009). Technical advances in robot-assisted laparoscopic radical prostatectomy. Ther Adv Urol.

[70] Su LM, Link RE, Bhayani SB, Sullivan W, Pavlovich CP (2004). Nerve-sparing laparoscopic radical prostatectomy: replicating the open surgical technique. Urology.

[71] Cao L, Yang Z, Qi L, Chen M (2019). Robot-assisted and laparoscopic vs open radical prostatectomy in clinically localized prostate cancer: perioperative, functional, and oncological outcomes: A Systematic review and meta-analysis. Medicine.

[72] Forsmark A, Gehrman J, Angenete E, Bjartell A, Björholt I, Carlsson S, Hugosson J, Marlow T, Stinesen-Kollberg K, Stranne J, Wallerstedt A, Wiklund P, Wilderäng U, Haglind E (2018). Health economic analysis of open and robot-assisted laparoscopic surgery for prostate cancer within the prospective multicentre LAPPRO trial. Eur Urol.

[73] Shkolyar E, Shih IF, Li Y, Wong JA, Liao JC (2020). Robot-assisted radical prostatectomy associated with decreased persistent postoperative opioid use. J Endourol.

[74] Geraerts I, Van Poppel H, Devoogdt N, Van Cleynenbreugel B, Joniau S, Van Kampen M (2013). Prospective evaluation of urinary incontinence, voiding symptoms and quality of life after open and robot-assisted radical prostatectomy. BJU Int.

[75] Coughlin GD, Yaxley JW, Chambers SK, Occhipinti S, Samaratunga H, Zajdlewicz L, Teloken P, Dunglison N, Williams S, Lavin MF, Gardiner RA (2018). Robot-assisted laparoscopic prostatectomy versus open radical retropubic prostatectomy: 24-month outcomes from a randomised controlled study. Lancet Oncol.

[76] Di Pierro GB, Baumeister P, Stucki P, Beatrice J, Danuser H, Mattei A (2011). A prospective trial comparing consecutive series of open retropubic and robot-assisted laparoscopic radical prostatectomy in a centre with a limited caseload. Eur Urol.

